# Exploring factors that influence HRQoL for people living with Parkinson’s in one region of Ireland: A cross-sectional study

**DOI:** 10.1186/s12877-022-03612-4

**Published:** 2022-12-23

**Authors:** Irene Cassidy, Owen Doody, Pauline Meskell

**Affiliations:** Department of Nursing and Midwifery, Faculty of Education and Health Sciences, Health Research Institute, Ageing Research Centre, University of Limerick, Limerick, Ireland

**Keywords:** Health-related quality of life (HRQoL), Quantitative, Survey, Parkinson’s

## Abstract

**Background:**

The diversity of symptoms associated with Parkinson’s and their impact on functioning have led to an increased interest in exploring factors that impact Health-Related Quality of Life (HRQoL). Although the experience of Parkinson’s is unique, some symptoms have a greater impact than others, e.g. depression. Moreover, as the risk of Parkinson’s increases with age, the financial and public health impact of this condition is likely to increase, particularly within the context of a globally ageing population. In Ireland, research is ongoing in the pursuit of causes and effective treatments for Parkinson’s; however, its impact on everyday living, functioning, and HRQoL is largely under-examined. This study aims to describe factors that influence HRQoL for people with Parkinson’s (PwP) in one region of Ireland.

**Methods:**

A cross-sectional postal survey was conducted among people living with Parkinson’s (*n* = 208) in one area of Ireland. This survey included socio-demographic questions, Nonmotor Symptoms Questionnaire for Parkinson’s disease (NMSQuest), the Geriatric Depression Scale (GDS-15), and the Parkinson’s disease Questionnaire (PDQ-39). Statistical analysis was conducted using SPSS, IBM version 25 (SPSS Inc., Chicago, II, USA).

**Results:**

Participants reflected a predominantly older population who were married, and lived in their own homes (91%). Participants diagnosed the longest reported poorer HRQoL regarding mobility, activities of daily living, emotional well-being, social support, cognition, communication domains and overall HRQoL. Lower HRQoL correlated with higher depression scores *p* < 0.001 and participants in the lower HRQoL cohort experienced 2.25 times more non-motor symptoms (NMSs) than participants with higher HRQoL. Hierarchical multiple linear regression analysis predicted Geriatric Depression Scale (GDS15) score, NMS burden, and years since diagnosis to negatively impact HRQoL. Principal component analysis (PCA) also indicated that for the population in this study, components measuring 1) independence/dependence 2) stigma 3) emotional well-being, and 4) pain were central to explaining core aspects of participants’ HRQoL.

**Conclusions:**

Findings highlighted the negative impact of longer disease duration, NMS burden, depression, mobility impairments, and perceived dependence on HRQoL for PwP. The positive influence of perceived independence, social engagement along with close supportive relationships were also identified as key components determining HRQoL. Findings emphasised the importance of long-term healthcare commitment to sustaining social and community supports and therapeutic, rehabilitative initiatives to augment HRQoL for PwP.

**Supplementary Information:**

The online version contains supplementary material available at 10.1186/s12877-022-03612-4.

## Background

Parkinsonism is a complex range of symptoms used to describe the motor features of resting tremor, bradykinesia, and muscular rigidity [1, 2]. The incidence of Parkinson’s is approximately 1.5 times higher in men compared with women [3], with delayed onset in women credited to the neuroprotective effects of oestrogen on the nigrostriatal dopaminergic system [2]. Parkinson’s is the second most common multifactorial neurodegenerative disorder after dementia [3], with an estimated 6.1 million people worldwide living with Parkinson’s [4]. In Ireland, the incidence is 1–2:1000 in the general population and 1:100 in those over 80. With a population of 4,761,865 [5] there is an estimated 12,000 people living with Parkinson’s in Ireland [6].

Idiopathic or classic Parkinson’s is the most common form of Parkinsonism, which is a slowly progressive neurodegenerative condition [7]. Parkinson’s pathology includes not only dopaminergic cells but also cholinergic, noradrenergic, serotonergic, histaminergic, and glutaminergic cells, explaining the wide clinical spectrum of features associated with the condition [8]. Consequently, Parkinson’s can result in progressive disability characterised by motor and nonmotor features [2] that have a significant impact on health [9]. Motor symptoms include shuffling gait and gait fenestration [1], facial masking, [10, 11], poor balance, and stooped posture [12]. Furthermore, difficulty standing and turning, freezing (brief inability to move) [13], ‘off periods’ [14] and speech that is low, slow, or difficult to understand [15] along with small handwriting are additional motor symptoms [1]. The clinical spectrum of Parkinson’s is much broader than a classical movement disorder. It encompasses many non-motor impairments like depression symptomology, anxiety, sleep disturbances, dementia, pain, constipation, swallowing ability (caused by sensory and saliva deficits), sexual, and urinary dysfunction [16, 17]. These non-motor symptoms are extremely prevalent and often present before the onset of motor symptoms [18].

Ageing remains one of the most significant risk factors for developing Parkinson’s [19]. As more people are living into old age, the overall number of people living with Parkinson’s is also set to rise. European estimates have predicted that Parkinson’s will double by 2030, primarily due to ageing populations [4, 20]. Living longer poses challenges for planning and provision of health services [21] that can respond to the needs of individuals living with chronic conditions, including those with, declining health, increasing healthcare requirements, loss of independence, and social isolation. Consequently, nurses and other healthcare professionals need to accommodate age-related changes, comorbidities, effects of long-term medication, possible cognitive decline, the impact of depression, anxiety, and caregiver support when leading and managing care for PwP [22]. Given that the complex interplay of factors influencing HRQoL in Parkinson’s predominantly occurs within the context of advancing age, it also strengthens the importance of planning for and delivering multifaceted interdisciplinary care to meet these individuals’ healthcare needs.

The concept of HRQoL normally indicates not only the influence of disease and treatment on disability and daily functioning but also the influence of perceived health on an individual’s capability to live a satisfying life [23, 24]. It is suggested that HRQoL is a broad, multidimensional concept that includes symptoms of disease or a health condition, treatment side effects, and functional status across physical, social, and mental health life domains [25]. Critical aspects of HRQoL include physical and sensory functioning, agility, cognition, pain, emotional and psychological well-being i.e., “all within the skin”, making it a multifaceted dynamic concept [26]. Evaluation of HRQoL helps us to understand a person’s condition, identify the main issues affecting HRQoL deterioration, and determine individual needs and priorities [23] to guide healthcare management decisions. Research findings have shown the impact of symptoms and predictors of HRQoL for PwP [27, 28]. For example, the specific impact of motor [1] and NMSs including anxiety and depression [29, 30], excessive daytime sleepiness [31], stigma [32] and multidisciplinary care [33] have been reported. From a nursing perspective, understanding the wide-ranging factors influencing HRQoL can focus assessments and support interventions to reduce the negative health-related impact of this chronic debilitating condition [34].

## Methods

### Aim and objectives

The aim of this study was to explore HRQoL for people living with Parkinson’s. This understanding is important to inform health policy, practice, education, and research so healthcare professionals in Ireland and internationally can respond effectively using person-centred approaches to support and care for PwP. The study objectives were to; i) describe demographic characteristics ii) describe types, and frequencies of NMSs, iii) investigate reported depression symptomology, iv) examine baseline HRQoL data (PDQ-39 scale), v) explore associations between socio-demographic variables, NMSs, depression symptomology, and perceived HRQoL, and vi) examine factors influencing HRQoL scores.

### Design

This research was part of a larger mixed methods study exploring QoL and living with Parkinson’s. A cross-sectional postal survey design using a self-reporting 91-item questionnaire across four scales was used in this study. Scale one, demographics, included 7 items consisting of gender, age, marital status, time since diagnosis, living arrangements e.g., where participants lived, whom participants lived with, and their sources of support. Scale two, the NMSQuest, consisted of 30 items focusing on participants’ accounts of NMSs with yes/no responses to each question [35, 36]. Scale three, GDS-15 [37], was used to draw attention to the presence of depression symptomology in the previous week, and responses required a yes/no answer [38]. Cut-off scores used in this study reflected suggestions of 0–4 as normal or no clinical concern, 5–8 mild depression symptomology, 9–11 moderate, and 12–15 severe depression symptomology [37]. Total scores were also recorded. Scale four, the PDQ-39, was included as a disease-specific, self-reporting HRQoL questionnaire, designed to assess aspects of functioning and well-being adversely affected by Parkinson’s [39]. The PDQ-39 is composed of 39 items across eight subscales/domains, including mobility, activities of daily living, emotional well-being, stigma, social support, cognition, communication, and bodily discomfort [39], and the instrument has tested strongly psychometrically [40]. The PDQ-39 Summary Index score (PDQ-39 SI) reflected the sum of dimension total scores divided by 8 [39]. The University Hospital Limerick Research Ethics Committee provided approval to undertake the survey. Informed consent was obtained; participants were informed of the study purpose, what participation would involve, the voluntary nature of participation, risks, benefits, confidentiality and links to further supports if needed.

### Participants and setting 

A purposive sample of the total population of people diagnosed with idiopathic Parkinson’s (*n* = 358) accessing neurological services at a University teaching hospital in one region of Ireland were invited to participate in this study. A response rate of 58.1% (*n* = 208) was achieved. Reasons for non-response included personal choice, health reasons, item nonresponse, or partial nonresponse (missing data). Data were collected within a PhD study completed in 2020.

### Statistical methods 

Data were analysed using data analysis software, Statistical Package for Social Sciences (SPSS), IBM version 25 (SPSS Inc., Chicago, II, USA). Demographics and NMSs: Descriptive analyses [41] including frequencies and mean rankings summarised participant characteristics and NMS data. GDS-15: descriptive analyses assessed scores and categories of depression symptomology. PDQ-39: Descriptive analyses initially calculated the mean, median, SD, frequencies,  range, and centile scores from each of the eight PDQ-39 dimensions and the PDQ-39 SI scores. Predominance of categorical and ordinal variables and violation of the assumption of normality evidenced by the Kolmogorov-Smirnov statistics [42] supported application of a range of non-parametric statistical tests (Additional file 1). The Mann-Whitney U was employed to test for differences between PDQ-39 SI scores and gender. The Kruskal-Wallis test analysed for differences between PDQ-39 SI scores, and age, years since diagnosis, NMS burden, and GDS-15 scores across three HRQoL cohorts (high, average, low). The relationships between number of NMSs (NMS burden) and PDQ-39 SI scores were investigated using Spearman’s rho test [42]. Odds ratios [43] analysed the proportion of respondents in each HRQoL cohort (high, average, low) likely to experience each of the 30 NMSs. Hierarchical multiple linear regression analysis determined the extent to which explanatory variables were linearly related to the PDQ-39 SI score. Finally, PCA was undertaken to produce linear combinations of PDQ-39 variables that explained most variability in correlation patterns [42].

## Results

### Participant characteristics

Three hundred and fifty-eight PwP were invited and 208 returned surveys (response rate of 58.1%). Of the respondents, 61.5% (*n* = 128) were men and 38.5% (*n* = 80) were women, 89.9% (*n* = 187) were over 60 years and 95.2% (*n* = 197) were diagnosed 2 years or longer. Over 72% (*n* = 150) were married, 70.7% (*n* = 147) lived with their spouse/partner and 90.9% (*n* = 189) lived in their own home. Mean rankings indicated husband, wife, or partner as the most important source of support (mean rank 3.34) (Table 1, Table 2).

**Table 1:** Participant characteristics

		N	%
Gender	Male	128	61.5
	Female	80	38.5
Age Category	20 or under	0	0
	21–30	0	0
	31–40	1	0.5
	41–50	1	0.5
	52–60	19	9.1
	61–70	62	29.8
	71–80	91	43.8
	81 or older	34	16.3
**Years since diagnosis**	1 yr or less	10	4.8
	2–6 yrs	90	43.5
	7–11 yrs	52	25.1
	12–16 yrs	34	16.4
	17–21 yrs	9	4.3
	22 yrs. or more	12	5.8
**Marital Status**	Married	150	72.1
	Widowed	31	14.9
	Divorced	5	2.4
	Separated	7	3.4
	Single	15	7.2
**Who - usually live with?**	Spouse/partner	147	70.7
	Alone	37	17.8
	Daughter	3	1.4
	Son	6	2.9
	Relative	3	1.4
	Friend	2	1.0
	Nursing home	7	3.4
	Other	3	1.4
**Where usually live**	Own home	189	90.9
	Partner’s home	1	0.5
	Relatives’ home	5	2.4
	Nursing home	7	3.4
	Other	6	2.9

**Table 2:** Sources of support

(Lowest mean = most important source of support)	Mean Rank
Husband, wife, or partner	3.34
Son or daughter	3.90
Friend or neighbour	6.66
Brother or sister	6.82
Home help	7.20
Professional carer	7.25
Son−/daughter in law	7.31
Other Family	7.73
Grandchild	7.80
Identified healthcare professionals	7.80
Other community services	7.93
Mother or father	7.94

### Types of NMSs

**Fig. 1:**
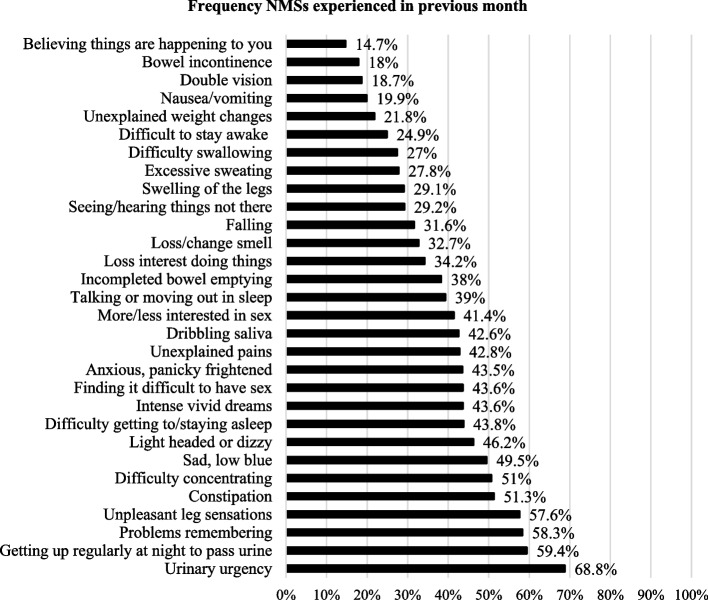
Types and Frequencies of NMSs reported by all participants

The top five NMSs identified were: a sense of urinary urgency 68.8% (*n* = 139), getting up regularly at night to pass urine 59.4% (*n* = 117), problems remembering or forgetting 58.3% (*n* = 119), unpleasant leg sensations 57.6% (*n* = 114) and constipation 51.3% (*n* = 102) (Fig. 1).

### Depression symptomology

GDS-15 results identified that 51.1% (*n* = 97) of respondents recorded normal scores or did not report symptomology of depression, 30% (*n* = 57) of participant scores indicated mild depression, 10.5% (*n* = 20) moderate depression, and 8.4% (*n* = 16) reported scores indicating severe depression. Of note, while 81.1% (*n* = 154) of respondents reported symptoms of either no or mild depression, the combined frequencies for moderate and severe depression symptomology was 18.9% (*n* = 36).

### HRQoL descriptives

The scores from each of the eight PDQ-39 dimensions along with the PDQ-39 SI score are presented in Additional file 2. In this study, the influence of movement and mobility alterations were reflected in the PDQ-39 survey data, where several respondents reported having trouble looking after their home (39.2%, *n* = 78) or getting around their house (27.7%, *n* = 56). Changes to movement, mobility, and bodily functioning also spanned beyond the home to community with some respondents reporting difficulty walking short distances (100 yards). There were 173 (83.2%) completed participant PDQ-39 SI scores that ranged from 0 to 72.76. The mean score was 30.34, median was 28.02, and standard deviation was 17.2. Higher PDQ-39 SI scores represented lower HRQoL and lower PDQ-39 SI scores represented higher HRQoL (Additional file 2).

### HRQoL and age

A Kruskal-Wallis test revealed a statistically significant difference in PDQ-39, dimension 1 ‘Mobility’ scores across age groups, (Gp 1, *n* = 0: 20 years or under, Gp 2, n = 0: 21–30 years, Gp 3 *n* = 1: 31–40 years, Gp 4 n = 0: 41–50 years; Gp 5, *n* = 17: 51–60 years, Gp 6, *n* = 57: 61–70 years, Gp 7, *n* = 86: 71–80 years, Gp 8, *n* = 26: 81 or older), X^2^ (4, *n* = 187) =12.4, *p* = 0.15, indicating that participants 81 years and older recorded a higher median score (Md = 60) than other age categories. Median values ranged from 2.5–47.5 suggesting that people in the oldest age category (81 years and older) had lower HRQoL in the mobility subscale of the PDQ-39 scale.

### HRQoL and years since diagnosis

Survey participants diagnosed the longest (22 years or more) scored poorer HRQoL in mobility *p* < 0.001, activities of daily living *p* < 0.001, social support *p* < 0.019, communication *p* < 0.001, and the overall PDQ-39 SI scores *p* < 0.001 than other years since diagnosis cohorts. Respondents diagnosed 7–11 years scored poorer HRQoL for emotional well-being than other years since diagnosis cohorts (*p* = 0.004), and participants diagnosed 12–16 years, and 22 years, reported poorer HRQoL with cognition (*p* = 0.008).

### HRQoL and NMS burden

The relationship between the PDQ-39 SI score and NMS burden was investigated using Spearman’s rho test. There was a strong positive correlation between the two variables, r = .660, *n* = 173, *p* < 0.001, with high levels of NMS burden associated with higher PDQ-39 SI scores meaning the higher the number of NMSs the lower the HRQoL.

Three even cohorts were created from the available 173 PDQ-39 SI scores. The first group represented people with higher HRQoL with PDQ-39 SI scores ranging from 0 to 19.69. The second cohort represented people with average HRQoL with scores ranging from 19.84–37.4. The third cohort included participants with lower HRQoL and scores ranging from 38.86–72.76. A Kruskal-Wallis test revealed a statistically significant difference in NMS burden scores across three HRQoL cohorts (high, average, low HRQoL), (Gp 1, *n* = 57: high HRQoL, Gp2, *n* = 56: average HRQoL, Gp3, *n* = 60: low HRQoL), X^2^ (2, *n* = 173) = 55.66, *p* < 0.001. The low HRQoL group recorded a higher median score (Md = 121.6) than the other two HRQoL cohorts (average HRQoL, Md = 85) and (high HRQoL, Md = 52.7) respectively, signalling that respondents in the low HRQoL cohort reported a higher NMS burden.

Respondents in the high HRQoL cohort reported experiencing 391 NMSs in the previous month. Those in the average HRQoL cohort reported experiencing 591 NMSs and those in the low HRQoL cohort reported 880 symptoms in the previous month. The NMS burden was 1.51 times higher for people in the average HRQoL cohort (average PDQ-39 SI scores) compared with those in the high HRQoL cohort (low PDQ-39 SI HRQoL scores). The NMS burden was 1.49 times higher for people in the low HRQoL cohort (low PDQ-39 SI scores) compared with those in the average HRQoL cohort (average PDQ-39 SI scores). Overall respondents in the low HRQoL cohort (high PDQ-39 SI scores) experienced 2.25 times more NMSs than respondents with high HRQoL.

### HRQoL cohorts and NMSs

**Fig. 2:**
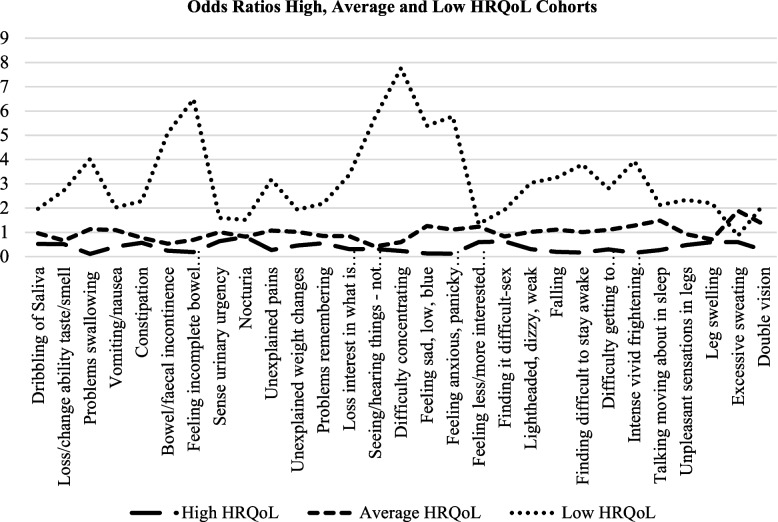
Odds ratios NMS and HRQoL cohorts

The odds ratios [41] for experiencing individual NMSs were calculated through examining the proportion of respondents in each HRQoL cohort, (high, average, and low HRQoL) likely to experience each of the 30 NMSs. As odds ratios can only be undertaken in a 2 by 2 table, three binary variables were used 1) ‘high’ HRQoL and ‘not high’ HRQoL 2) ‘average’ HRQoL and ‘not average’ HRQoL and 3) ‘low’ HRQoL and ‘not low’ HRQoL. The odds of NMSs such as difficulty concentrating (OR 7.77), incomplete bowel emptying (OR 6.49), feeling anxious (OR 5.78), seeing or hearing things that others say are not true (OR 5.73) were more likely to be experienced by participants with lower HRQoL (than participants in high and average HRQoL cohorts) (Fig. 2).

### HRQoL cohorts and depression symptomology

**Fig. 3:**
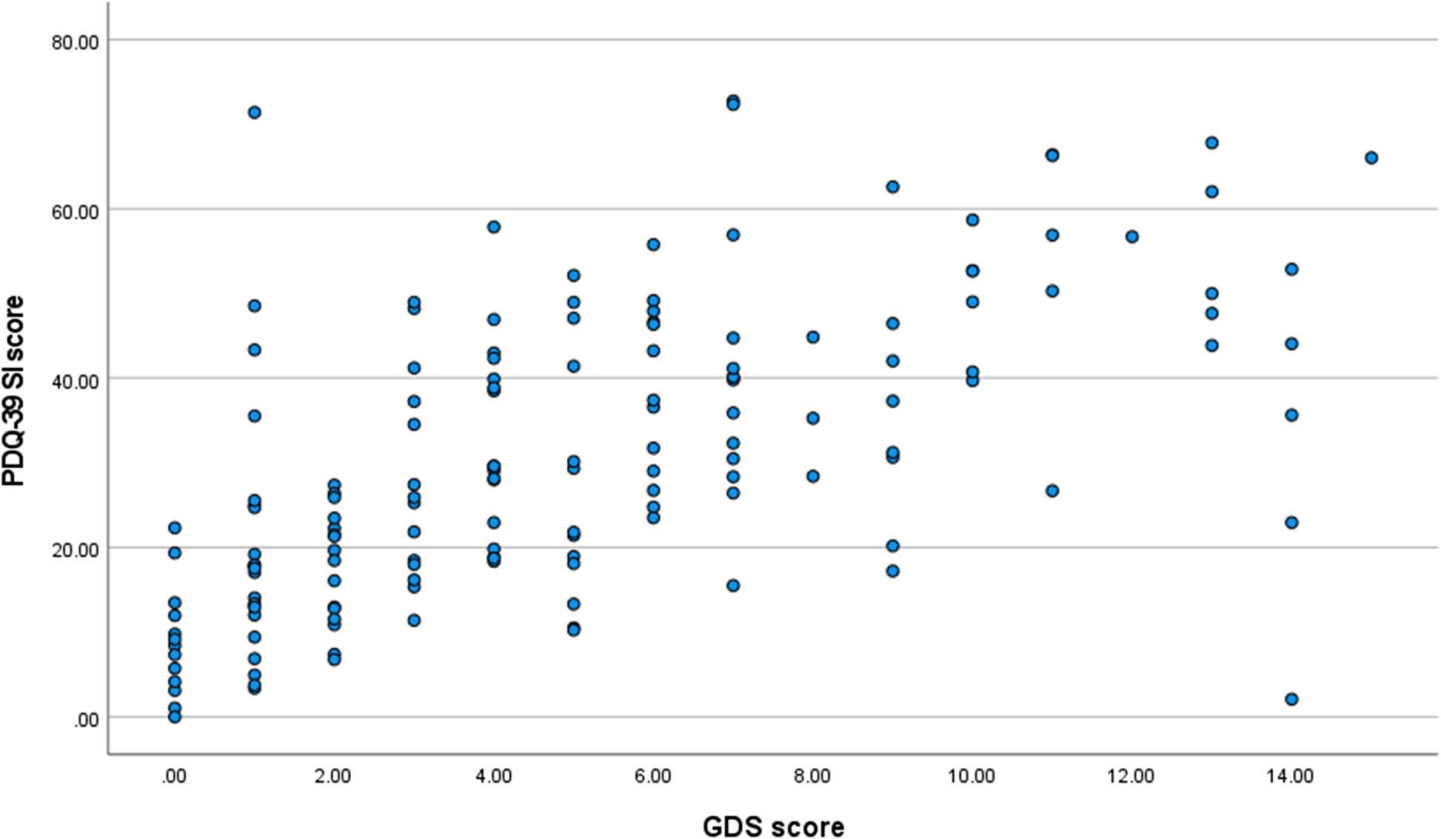
Spearman’s rho- Correlation GDS-15 scores and PDQ-39 SI scores

A Kruskal-Wallis test revealed a statistically significant difference in GDS-15 scores across the three HRQoL cohorts (high, average, low HRQoL), Gp 1, *n* = 52: high HRQoL, Gp2, *n* = 50: average HRQoL, Gp3, *n* = 55: low HRQoL), X^2^ (2, *n* = 157) = 60.13, *p* < 0.001. The low HRQoL group recorded a higher median score (Md = 7) than the other two HRQoL cohorts which recorded medians of 4 (average HRQoL) and 1 (high HRQoL). These results indicate that respondents in the lower HRQoL cohort reported higher GDS-15 scoring for depression symptomology. The relationship between overall HRQoL scores (PDQ-39 SI scores) and depression symptomology scores (GDS-15) utilising Spearman’s rho identified a strong positive correlation between the two variables rho = 0.609, *n* = 177, *p* < 0.001, with high PDQ-39 SI scores associated with high depression scores (higher PDQ-39 SI scores indicate poorer HRQoL) (Fig. 3).

### Assessing predictors of HRQoL

Hierarchical multiple regression was used to assess the ability of four control measures (gender, where participants usually lived, who they usually lived with, and marital status) to predict overall HRQoL (as measured by the PDQ-39 SI score), after controlling for the influence of the GDS-15 score, NMS burden, years since diagnosis and age. Preliminary analyses ensured no violation of the assumptions of normality, linearity, multicollinearity, and homoscedasticity. GDS-15 score, NMS burden, years since diagnosis, and age were entered at step 1 explaining 53.9% of the variance in HRQoL, F (4, 152) = 44.37, *p* < 0.001.

After entry of the variables where participants usually lived, who they usually lived with, marital status, and gender at step 2, the total variance explained by the model was 56.9%, R^2^ change = .030, F (8, 148) = 24.43, F change = 2.61, *p* = .038. Hence, the second block of control measures only explained a further 3% of the variance in HRQoL after controlling for GDS-15 score, NMS burden, years since diagnosis and age. These results supported model 1 as the model that most accurately explained the effects of key variables including demographics, NMSs and reported depression symptomology on participants’ HRQoL.

Model 1 with four variables GDS-15 score, NMS burden, years since diagnosis, and age were predicted to negatively influence HRQoL. Three of the four control variables were statistically significant. The NMS burden measure recorded the highest beta value (beta = 0.415, *p* < 0.001) followed by the GDS-15 measure (beta = 0.374 *p* < 0.001). The lowest significant beta measure was years since diagnosis (beta −.151 *p* = 0.008). These results strengthen understanding around the impact of depression symptomology, the number of NMSs experienced and length of time diagnosed, in predicting HRQoL for individuals living with Parkinson’s (Additional File 3).

### Underlying components of HRQoL

The 39 items of the PDQ-39 underwent PCA to investigate underlying components of the data. The Kaiser-Meyer-Olkin measure of sampling adequacy was 0.91, and Bartlett’s test of sphericity was significant (*p* < 0.001) confirming that the observed correlation matrix was appropriate for PCA. Initial analysis revealed the presence of seven components with eigenvalues exceeding 1, explaining 65.5% of total variance. Examination of the rotated component matrix indicated the removal of eight questions from the analysis that had patterns of low coefficient loadings < 0.6.

Analysis was rerun with six components explaining 67.9% variance; four factors accounted for 60.2% of this and the screeplot revealed a clear break after four components. Results from parallel analysis also showed four components with eigenvalues exceeding the corresponding criterion values for a randomly generated data matrix of the same size (39 variables X 208 participants). Hence, a four-component solution was forced. To aid interpretation of these four components oblique rotation (varimax) was performed. The rotated solution showed loadings on four components accounting for 60.2% variance, component 1 accounted for 40.2%, component 2 a further 8.49%, component 3, 6.13%, and component 4, 5.37% variance. This analysis strengthens recognition of the multidimensional influences on HRQoL for PwP including perceptions of independence/dependence, stigma stemming from interactions with others, emotional well-being, and NMSs such as pain (Table 3).

**Table 3:** Principal Component Analysis

Component	Title	PDQ-39 Underlying Latent Variables
1	**Independence/dependence** This component incorporated all 13 questions from the first two dimensions of the PDQ-39 scale; Mobility and Activities Daily Living (ADL). The essence of this component reflects participants’ views around the HRQoL impact of Parkinson’s on perceived independence/dependence.	
		PDQ-39 Mobility dimension (Q1-Q10) PDQ-39 ADL dimension (Q11-Q13)
2.	**Experiencing stigma** This component incorporated 2 questions from the PDQ-39 Communication dimension and 2 from the original Stigma dimension. This component elucidates the impact of perceived stigma, particularly when communicating and interacting with other people on participants’ HRQoL	
		PDQ-39 Communication dimension (Q34-Q35) PDQ-39 Stigma dimension (Q25, Q26)
3	**Emotional well-being** This component incorporated five questions from the PDQ-39 Emotional well-being dimension and explains patterns relating to the influence of emotional well-being on participants’ HRQoL.	
		PDQ-39 Emotional well-being dimension (Q17-Q20, Q22)
4	**Experiencing pain** This component included 2 questions from the Bodily discomfort dimension and reflects the impact of painful experiences such as cramps, spasms, aching/painful joints or body on participants’ HRQoL.	
		PDQ-39 Bodily discomfort dimension (Q37 and Q38)

## Discussion

Respondents included a predominantly older population who were married, lived with their spouse/partner in their own home, and had been diagnosed with Parkinson’s for two years or longer. Consistent with international literature, there were more men than women in the sample [44]. Interestingly, a large proportion of survey respondents in this study reported never having problems with close personal relationships or lacking support from family or close friends. Indeed, a profile of people living with Parkinson’s for over 20 years showed that the majority lived at home with a family caregiver [45]. Elsewhere, “social relationships” (with family, friends, and neighbours) were revealed as most significant for PwP [46]. Karlstedt et al. [47] found that although PwP may deteriorate over time, high relationship quality can produce gratification, meaning, and support, enhance negative consequences, and improve HRQoL. This emphasises the importance of establishing a profile of available supports, to guide and facilitate pathways for individualised person-centred care [48], that can support the HRQoL of PwP. Hence, interventions embracing social support, respite care, couple therapy, or counselling may help caregivers and individuals adapt and adjust to ever-changing care situations and find inward strength to cope [47].

In this study, presence, type, and disruptions caused by NMSs, often referred to as invisible symptoms, manifested their influence on HRQoL. Concurring reports have suggested that NMSs influence long-term HRQoL for PwP [49]. Moreover, findings show how they may influence HRQoL more than motor symptoms [40], and psychological well-being [50] thereby highlighting the impact of NMSs and their relationship to HRQoL.

Within this study, respondents living with Parkinson’s for longer timeframes, recorded a higher NMS burden, a finding supported by Sanchez-Martinez et al. [17]. There was also a greater than two-fold increase in the NMS burden for those in the low HRQoL cohort compared with participants in the high HRQoL cohort. Results indicated that the odds of experiencing individual NMSs were more likely in participants with lower HRQoL. Indeed, NMS burden can negatively affect HRQoL [51]. Furthermore, people with more severe NMSs have reported more unmet needs such as accessibility to healthcare professionals [22]. Given that respondents were community-dwelling, this highlights the vital role of primary and community nurses in providing impeccable assessment and therapeutic care that alleviates the impact of NMSs on PwP [22].

As detailed in the results, most survey respondents reported either no depression or mild depression; nonetheless, combined frequencies for moderate and severe depression symptomology were clinically significant. This supports earlier findings [52, 53] signalling the prevalence of major depressive disorders in PwP. Principal component and regression analyses established depression score as a significant contributor to, and determinant of HRQoL, which is comparable with previous research results that highlighted depression as one of the most important determinants of poor HRQoL for PwP [27, 54].

Of interest within this study was the finding which revealed a statistically significant difference in HRQoL scores (high, average, low HRQoL cohorts) across GDS-15 depression symptomology categories. Respondents in the low HRQoL cohort recorded more severe depression symptomology. From a health-professional perspective, two simple questions are recommended to assist identification of depression symptomology in the previous month, ‘have you often been bothered by feeling down, depressed or hopeless’? and, ‘Have you often been bothered by having little interest or pleasure in doing things’? [55] Undoubtedly, being vigilant to depression symptomology, employing listening skills, along with ascertaining a comprehensive history are fundamental nursing skills. Furthermore, referral to healthcare practitioners competent to undertake more in-depth physical and mental health assessments using validated instruments (e.g., GDS-15 or the combined Hospital Anxiety and Depression Scale HADS) is advised to indicate presence and severity of psychological difficulties, identify individual preferences, along with family, and social support needs [56, 57]. This is particularly important given the negative impact of depression and anxiety on the lives of PwP [58].

Findings from this study also emphasised the everyday impact of Parkinson’s on ambulation, where almost half (48.5%) of respondents reported difficulty walking ½ mile. Findings from the wider literature highlight that exercise may help motor and NMSs and prevent secondary complications of immobility (cardiovascular, osteoporosis) [59]. Additionally, exercise can help optimise function and performance of life activities [60]. Developing exercise and walking capacity, balance, muscle power, and compensatory methods to alleviate pain during ambulation could also reduce the work of walking from a rehabilitation perspective [61]. Through rehabilitation, growth in knowledge and skills can increase personal control, self-management, role participation, and satisfaction [62]. This stresses the importance of resource planning and long-term healthcare professional commitment to sustaining rehabilitative therapy initiatives for PwP that truly foster independence and augment HRQoL.

However, in this study, the influence of Parkinson’s on mobility went beyond mere estimates of walking distance; many participants indicated they would need someone to accompany them in public. Furthermore, almost a third of survey participants reported that they felt frightened (often or always) about falling over in public. Horning et al. [63] reported gait deterioration, falls, and functional decline as key symptoms challenging home safety. These cause noticeable restrictions in a range of activities, disability, and altered autonomy [64] and highlight the interwoven nature of mobility, fear, and dependence. Falls are significantly related to reduced HRQoL [34, 65]. More recently, it was reported that ‘locomotion dysfunction’ (gait and balance difficulties) scored highest in motor characteristics underscoring the importance of gait deficits to HRQoL [28]. One common dysfunction, freezing of gait, is a complex motor fluctuation connected with advancing Parkinson’s [66]. As motor fluctuations also have direct and indirect effects on HRQoL (directly affecting pain and fatigue, and indirectly mood and sleep), assessing and managing these effectively could improve overall HRQoL [67]. Recommendations from this study include the need to supplement HRQoL research instruments such as the PDQ-39 by including additional instruments to capture the everyday impact of Parkinson’s such as freezing of gait (e.g. The Freezing of Gait Questionnaire FoGQ), and other influences such as falls incidence and fear of falling (FoF) e.g. Fall Efficacy Scale-International (FES-I).

Principal component analysis in this study (PDQ-39 scale) revealed one of the four components determining HRQoL was independence/dependence. Previously, perceived autonomy was reported to affect HRQoL [68]. Being dependent on others causes feelings of insecurity and reduces confidence in self-image and identity [69]. The idea that Parkinson’s impacts meaningful activities was previously linked to losses associated with previous social identities such as gardener, cook, mother/father, or being able to wear heeled shoes [70]. Without sufficient personal control, people also find adaptation problematic [71]. Subsequently, they may experience increased effects of chronic illness and its treatment, resulting in even lower HRQoL. Findings from Gusdal et al.’s [72] delphi study described having a social life, feeling safe and being able to retain one’s control as requirements for healthy and independent living among older people generally. Social care is valued for allowing choice, control, and enabling people to maintain independence [73]. Hence, more creative community supports, based on individual needs in line with an active ageing concept [74], are required to facilitate social and advocacy needs around independence to sustain and improve HRQoL. This is further strengthened by aspirations to support people to live independently in the community for as long as possible, and to co-design population service requirements [21]. Udo’s (2016) [75] analysis of active ageing also incorporates continued participation in life and health to enable individuals to enjoy a positive QoL, as they grow older. Hence, promotion of independence and participation must be primary considerations for healthcare policy development and service delivery to preserve HRQoL for PwP.

Survey findings indicated over a third of respondents had difficulty getting around in public, and/or needed someone to accompany them. Over half the respondents indicated a preference for staying at home rather than socialising. In addition, poorer HRQoL in the domain of social support was evidenced by respondents with longstanding Parkinson’s. Social isolation independently influences the prospect of depression or anxiety, with loneliness being a cyclical risk factor for declining social life [76]. All these situations ultimately reduce social roles, well-being, and HRQoL.

While the largely inevitable physical consequences of disease take hold, social withdrawal and isolation should not be viewed as inevitable nor intractable [76]. From a health and social perspective, activities that engage PwP where they feel valued, and part of communities is essential. This is especially important for individuals diagnosed for longer periods, where social isolation may creep in. This again emphasises the importance of addressing loneliness and social network size to improve mental health of older PwP [76]. It is vital that barriers to participation are removed for older people generally and more opportunities emerge for continued involvement in cultural, economic, and social dimensions of life, according to needs, preferences, and capabilities [21]. From a HRQoL perspective, healthcare professionals should be mindful of the positive influence of feeling part of a community of people, be that a committee or a group.

### Limitations and recommendations

This research was conducted in one area of Ireland, therefore participants’ responses may not be generalisable to the whole population of people living with Parkinson’s. The survey reflected self-reports at a single point in time with no follow-up survey. The stage of Parkinson’s was not clinically assessed during the research, hence, progression patterns and severity could not be objectively assessed and subsequently related to key research variables and subjective reports of HRQoL. Missing data was omitted from the analysis.

The NMS Quest, and GDS-15 described presence of symptoms and the PDQ-39 assessed aspects of functioning and well-being adversely affected by Parkinson’s. However, these instruments did not examine participants’ experiences and feelings around NMSs, depression and HRQoL. This calls for more in-depth qualitative exploration to expand breadth and range of inquiry and provide fuller and deeper understandings on the impact of Parkinson’s on participants’ lives.

Focused multidisciplinary rehabilitative programmes targeting walking ability, balance, FoG, falls and FoF should be available in primary care to prevent and address sedentary lifestyle, and falls. It is also recommended that the PDQ-39 as a Parkinson’s specific HRQoL instrument is revised to capture the everyday impact of Parkinson’s on individuals’ lives such as FoG, falls, and FoF. Future research should consider sampling and recruitment within the context of gender representation to identify if gender sensitive healthcare interventions may be effective in ameliorating the impact of NMSs on perceived HRQoL. Healthcare policy needs to keep promotion of independence as a central consideration when resourcing service delivery for PwP. This is important to support people to live independently in their own community for as long as possible.

## Conclusions

This study aimed to explore factors that influence HRQoL for PwP. From a broader perspective, the findings allude to the positive benefits of close relationships in providing a supportive context moderating the psychological well-being of participants**,** particularly within the context of living in one’s own home. Overall, findings indicated the differing impact of NMSs on HRQoL of men and women, those with longer disease duration, and people with poorer self-reported HRQoL. The impact of depression was identified, stressing the need for vigilance, comprehensive assessment, and referral for individualised follow-up care. This is important given the evidence illuminating the powerful influence of depression on overall QoL in Parkinson’s. The influence of movement and mobility alterations were reflected in the PDQ-39 survey data, which also revealed Independence/dependence and social engagement as key components determining HRQoL for PwP.

These findings strengthen recognition of HRQoL determinants as more than just physical but as also encapsulating a sense of self, emotional well-being, feelings of control, and social engagement. Statistical analysis in this study’s population also uniquely established a framework of regression that reinforces understanding around the impact of depression symptomology, NMSs, and the length of time diagnosed in predicting HRQoL for PwP. These findings stress the importance of resource planning and long-term healthcare professional commitment to sustaining rehabilitative therapy initiatives for PwP that foster independence and augment HRQoL.

## Supplementary Information

**Additional file 1.** Kolmogorov-Smirnov Statistics. Details-Portrait presentation of results.

**Additional file 2.** HRQoL descriptives. Details- Landscape presentation of results.

**Additional file 3.** Hierarchical multiple regression analysis. Details- Landscape presentation of results.

## Data Availability

The datasets used and analysed during the current study are not publicly available as participants have not given consent for public availability of their data. But it is available from the corresponding author on reasonable request.

## Abbreviations

FoF: Fear of Falling
FoG: Freezing of Gait
GDS-15: Geriatric Depression Scale- 15 items
HRQoL: Health-Related Quality of Life
NMSQuest: Nonmotor Symptoms Questionnaire for Parkinson’s disease
PDQ: Parkinson’s disease Questionnaire
PwP: People with Parkinson’s
