# Supplementary material for: Exploring factors that influence HRQoL for people living with Parkinson’s in one region of Ireland: A cross-sectional study
Source: BMC Geriatr. 2022 Dec 23;22:994. doi: 10.1186/s12877-022-03612-4 (PMC9784292; doi:10.1186/s12877-022-03612-4)
Supplement: Supplementary file 1 — Additional file 1. Kolmogorov-Smirnov Statistics. Details-Portrait presentation of results. [file 12877_2022_3612_MOESM1_ESM.docx]

**Additional File 1- Kolmogorov-Smirnov statistics**

| **Kolmogorov-Smirnov statistics** | | | | | | |
| --- | --- | --- | --- | --- | --- | --- |
|  | Kolmogorov-Smirnov^a^ | | | Shapiro-Wilk | | |
|  | Statistic | Df | Sig. | Statistic | Df | Sig. |
| GDS-15 Score | .119 | 177 | .000 | .928 | 177 | .000 |
| PDQ-39 Dim 1 | .084 | 187 | .003 | .943 | 187 | <.001 |
| PDQ-39 Dim 2 | .130 | 194 | <.001 | .926 | 194 | <.001 |
| PDQ-39 Dim 3 | .126 | 187 | <.001 | .940 | 187 | <.001 |
| PDQ-39 Dim 4 | .181 | 199 | <.001 | .863 | 199 | <.001 |
| PDQ-39 Dim 5 | .272 | 193 | <.001 | .701 | 193 | <.001 |
| PDQ-39 Dim 6 | .123 | 193 | <.001 | .957 | 193 | <.001 |
| PDQ-39 Dim 7 | .163 | 197 | <.001 | .889 | 197 | <.001 |
| PDQ-39 Dim 8 | .122 | 200 | <.001 | .962 | 200 | <.001 |
| PDQ-39 SI | .077 | 173 | .014 | .969 | 173 | <.001 |
