# Supplementary material for: Exploring factors that influence HRQoL for people living with Parkinson’s in one region of Ireland: A cross-sectional study
Source: BMC Geriatr. 2022 Dec 23;22:994. doi: 10.1186/s12877-022-03612-4 (PMC9784292; doi:10.1186/s12877-022-03612-4)
Supplement: Supplementary file 2 — Additional file 2. HRQoL descriptives. Details- Landscape presentation of results. [file 12877_2022_3612_MOESM2_ESM.docx]

**Additional File 2- HRQoL descriptives**

| PDQ-39 Descriptive statistics | | | | | | | | | | |
| --- | --- | --- | --- | --- | --- | --- | --- | --- | --- | --- |
| **Dimension** | | **n** | **Mean** | **Median** | **SD** | **Variance** | **Range** | **Centile 25** | **Centile**  **50** | **Centile**  **75** |
| Dimension 1 | Mobility | 187 | 45.01 | 42.5 | 30.87 | 953 | 100 | 17.5 | 42.5 | 72.5 |
| Dimension 2 | ADL | 194 | 41.66 | 37.5 | 31 | 964 | 100 | 12.5 | 37.5 | 67.7 |
| Dimension 3 | Emotion | 187 | 28.9 | 25 | 21.99 | 483 | 100 | 12.5 | 25 | 45.8 |
| Dimension 4 | Stigma | 199 | 18.4 | 12.5 | 19.43 | 377.68 | 87.5 | 0 | 12.5 | 31.25 |
| Dimension 5 | Social Support | 193 | 12.3 | 0 | 18.8 | 354 | 100 | 0 | 0 | 16.7 |
| Dimension 6 | Cognition | 193 | 31.7 | 31.25 | 21.19 | 449.4 | 87.5 | 12.5 | 31.25 | 50 |
| Dimension 7 | Communication | 197 | 24.3 | 16.67 | 23 | 531.7 | 100 | 0 | 16.67 | 33.33 |
| Dimension 8 | Bodily discomfort | 200 | 37.9 | 33.33 | 23.7 | 562 | 100 | 25 | 33.33 | 50 |
| SI score | SI | 173 | 30.3 | 28 | 17.2 | 296 | 72.76 | 17.4 | 28 | 43.6 |
