# Supplementary material for: Exploring factors that influence HRQoL for people living with Parkinson’s in one region of Ireland: A cross-sectional study
Source: BMC Geriatr. 2022 Dec 23;22:994. doi: 10.1186/s12877-022-03612-4 (PMC9784292; doi:10.1186/s12877-022-03612-4)
Supplement: Supplementary file 3 — Additional file 3. Hierarchical multiple regression analysis. Details- Landscape presentation of results. [file 12877_2022_3612_MOESM3_ESM.docx]

**Additional File 3 Hierarchical Multiple Regression Analysis**

**Regression model summary**

| **Model** | **R** | **R square** | **Adjusted R Square** | **Std. Error**  **Estimate** | **Change Statistics** | | | | |
| --- | --- | --- | --- | --- | --- | --- | --- | --- | --- |
|  |  |  |  |  | **R Square Change** | **F Change** | **df1** | **df2** | **Sig. F Change p<** |
| 1 | .734^a^ | .539 | .527 | 11.83959 | .539 | 44.373 | 4 | 152 | .001 |
| 2 | .754^b^ | .569 | .546 | 11.59645 | .030 | 2.610 | 4 | 148 | .038 |

ANOVA results

| **Model** | | **Sum of Squares** | **df** | **Mean Square** | **F** | **Sig.**  **P<** |
| --- | --- | --- | --- | --- | --- | --- |
| 1 | 1. Regression | 24880.190 | 4 | 6220.047 | 44.373 | .001 |
|  | Residual | 21306.722 | 152 | 140.176 |  | .001 |
|  | Total | 46186.912 | 156 |  |  |  |
| 2 | 1. Regression | 26284.221 | 8 | 3285.528 | 24.432 | .001 |
|  | Residual | 19902.692  19902.692 | 148 | 134.478 |  |  |
|  | Total | 46186.912 | 156 |  |  |  |
| 1. **Dependent variable :** PDQ-39 SI (total score). 2. **Predictors: (Constant),** Age<80 or 81 and older, Years since Diagnosis < 11 years or 12 years and older, GDS-15 score, NMS Burden. c. Predictors: (Constant) Age<80 or 81 and older, Years since diagnosis <11 years or 12 years and older, GDS-15 score, NMS burden, Gender, Usually lives own home/doesn’t usually live own home, Usually lives spouse/partner/doesn’t usually live spouse/partner, Married/not currently married | | | | | | |

Coefficients

| **Model** | | **Unstandardised coefficients** | | **Standardised coefficients** | **t** | **Sig.**  **P<** | **Correlations** | | | **Collinearity Statistics** | |
| --- | --- | --- | --- | --- | --- | --- | --- | --- | --- | --- | --- |
|  |  | **B** | **Std. Error** | **Beta** |  |  | **Zero-order** | **Partial** | **Part** | **Tolerance** | **VIF** |
| 1 | (Constant) | 12.026 | 2.843 |  | 4.229 | .001 |  |  |  |  |  |
|  | GDS-15 score | 1.679 | .292 | .374 | 5.746 | .001 | .609 | .422 | .317 | .718 | 1.393 |
|  | NMS burden | 1.256 | .198 | .415 | 6.354 | .001 | .639 | .458 | .350 | .711 | 1.406 |
|  | Years since diagnosis <11years or 12 years & older, | -5.858 | 2.175 | -.151 | -2.694 | .008 | -.270 | -.213 | -.148 | .969 | 1.032 |
|  | Age <80 or 81 & older | 3.228 | 2.568 | .070 | 1.257 | .211 | .074 | .101 | .069 | .992 | 1.008 |
| 2 | (Constant) | 16.017 | 9.829 |  | 1.629 | .105 |  |  |  |  |  |
|  | GDS-15 score | 1.626 | .291 | .362 | 5.579 | .001 | .609 | .417 | .301 | .692 | 1.445 |
|  | NMS burden | 1.247 | .196 | .412 | 6.355 | .001 | .639 | .463 | .343 | .693 | 1.443 |
|  | Years since diagnosis <11years or 12 years & longer | -6.858 | 2.156 | -.176 | -3.182 | .002 | -.270 | -.253 | -.172 | .946 | 1.057 |
|  | Age<80 or 81 & older | 1.759 | 2.557 | .038 | .688 | .493 | .074 | .056 | .037 | .960 | 1.042 |
|  | Usually/does not usually live with spouse/partner | -4.383 | 4.678 | -.116 | -.937 | .350 | -.179 | -.077 | -.051 | .189 | 5.287 |
|  | Usually/does not usually live in own home | -2.021 | 3.567 | -.034 | -.567 | .572 | -.160 | -.047 | -.031 | .812 | 1.231 |
|  | Gender | -.208 | 2.024 | -.006 | -.103 | .918 | -.015 | -.008 | -.006 | .885 | 1.130 |
|  |  |  |  |  |  |  |  |  |  |  |  |
|  | Married/not currently married | 2.013 | 4.665 | .053 | .431 | .667 | .163 | .035 | .023 | .196 | 5.102 |

a. Dependent Variable : PDQ-39 SI (Total Score)
